# Directed carbapenemase testing is no longer just for Enterobacterales: cost, labor, and workflow assessment of expanding carbapenemase testing to carbapenem-resistant *P. aeruginosa*

**DOI:** 10.1080/22221751.2023.2179344

**Published:** 2023-03-01

**Authors:** Christian M. Gill, Poonam Rajkotia, Amity L. Roberts, Fred C. Tenover, David P. Nicolau

**Affiliations:** a Center for Anti-Infective Research & Development Hartford Hospital, Hartford, CT, USA; b Microbiology Laboratory Services, Hartford Healthcare Ancillary Microbiology Laboratory, Newington, CT, USA; c Cepheid, Sunnyvale, CA, USA; d Department of Infectious Diseases, Hartford Hospital, Hartford, CT, USA

**Keywords:** Carbapenemase, *P. aeruginosa*, Enterobacterales, workflow, rapid diagnostics testing

## Abstract

Molecular carbapenem-resistance testing, such as for the presence of carbapenemases genes, is commonly implemented for the detection of carbapenemase-producing Enterobacterales. Carbapenemase-producing *P. aeruginosa* is also associated with significant morbidity and mortality, although; prevalence may be underappreciated in the United States due to a lack of carbapenemase testing. The present study sought to compare hands-on time, cost and workflow implementation of carbapenemase gene testing in Enterobacterales and *P. aeruginosa* isolates versus sending out isolates to a public health laboratory (PHL) for testing to assess if in-house can provide actionable results. The time to carbapenemase gene results were compared. Differences in cost for infection prevention measures were extrapolated from the time of positive carbapenemase gene detection in-house versus PHL. The median time to perform carbapenemase gene testing was 7.5 min (range 5–14) versus 10 min (range 8–22) for preparation to send isolates to the PHL. In-house testing produced same day results compared with a median of 6 days (range 3–14) to receive results from PHL. Cost of in-house testing and send outs were similar ($46.92 versus $40.53, respectively). If contact precautions for patients are implemented until carbapenemase genes are ruled out, in-house testing can save an estimated $76,836.60 annually. Extension of in-house carbapenemase testing to include *P. aeruginosa* provides actionable results 3–14 days earlier than PHL Standard Pathway testing, facilitating guided therapeutic decisions and infection prevention measures. Supplemental phenotypic algorithms can be implemented to curb the cost of *P. aeruginosa* carbapenemases testing by identifying isolates most likely to harbour carbapenemases.

## Introduction

Carbapenemase-producing Enterobacterales (CP-CRE) are recognized as a clinical challenge since therapeutic options are limited and infections with such organisms are associated with significant mortality[1,2]. Prompt antimicrobial therapy active against *Klebsiella pneumonia*e carbapenemase (KPC)-producing Enterobacterales (KPC CP-CRE) has been associated with reduced mortality[3]. Specifically, novel-β-lactam-β-lactamase-inhibitor combinations (i.e. ceftazidime/avibactam) have been shown to be highly effective for management of KPC CP-CRE infections[3]. The introduction of rapid molecular carbapenemase gene detection has been associated with a reduction in both the time to appropriate therapy and mortality when results are reported to clinicians to act upon[4,5]. Molecular carbapenemase tests often detect the more prevalent carbapenemase genes, including KPC, New Delhi Metallo-β-lactamase (NDM), Verona Integron-Encoded Metallo-β-lactamase (VIM), Imipenemase (IMP), and Oxacillinase-48 (OXA-48). While carbapenemase genes are more frequently tested in Enterobacterales, these genetic elements have also been identified in *Pseudomonas aeruginosa* and *Acinetobacter* species. Carbapenemase-producing, carbapenem-resistant *P. aeruginosa* (CP-CRPA) is a notable threat globally and infections caused by CP-CRPA are associated with high morbidity and mortality[6,7]. Although porin loss and enhanced efflux systems account for the majority of CRPA in the United States, there is significant regional variation in the number of laboratories that undertake carbapenemase genes detection to identify CP-CRPA [6,8]. Thus, there may be an under appreciation of carbapenemase production in multi-drug resistant *P. aeruginosa* in the United States as carbapenemase gene testing is less commonly undertaken[9].

Several phenotypic carbapenemase-testing methods are available for *P. aeruginosa* testing including the CarbaNP test and the modified carbapenem inactivation method (mCIM) as described by the Clinical and Laboratory Standards Institute (CLSI)[7,10]. CarbaNP and mCIM tests are useful for alerting clinicians to the presence of a carbapenemase gene in CP-CRPA, however, these supplemental tests are unable to differentiate between serine-based and metallo- carbapenemases, which is important information for selecting antimicrobial therapy[6,10,11]. Phenotypic tests, especially mCIM, are limited by an extended turn-around time with ∼24 h of incubation required to obtain results [10]. Both the extended time to results and lack of carbapenemase class differentiation limit the utility of phenotypic carbapenemase testing for timely targeted antimicrobial therarpy [12]. Since clinical laboratories are typically mandated to submit CRE and carbapenem-resistant *Acinetobacter baumannii* (CRAB) isolates to their state/local public health laboratories (PHL) as part of the Laboratory Reports of Significant Findings, local testing to detect carbapenemases genes is often deferred because these services are provided. The Antimicrobial Resistance Network (ARN) also provides testing and tracking capacity for multidrug-resistant organisms around the country [13]. It is unclear, though, if relying on PHL Standard Pathway-generated results, which may take up to a week to be received, negatively impacts time to effective treatment and the initiation of infection prevention measures to interrupt transmission of the pathogens in the hospital.

The introduction of FDA-approved tests for carbapenemases genes has enabled clinical microbiology laboratories to differentiate between serine and metallo-β-lactamase-producing bacterial species often in less than one hour [14]. Implementation has been simplified by sample-to-answer formats, high throughput instrumentation options, and high analytical assay performance. Turnaround times will vary by modality, with next-generation sequencing (NGS) options requiring considerably more time versus rapid commercial tests run in-house. The commercial tests typically detect multiple carbapenemases gene classes [14]. The uptake of these tests has been widely adopted for Enterobacterales but testing of *P. aeruginosa* and *A. baumannii* isolates has been more limited [9,14,15]. One limitation of molecular carbapenemase test implementation for *P. aeruginosa* in particular is the cost of the tests since a high proportion of CRPA are due to resistance mechanisms other than carbapenemases[8]. As such, CP-CRPA may be underappreciated due to the lack of carbapenemase gene testing[9]. Previous phenotypic testing algorithms using results from a limited number of antimicrobial agents have been validated for identifying the CRPA isolates most likely to harbour carbapenemases. Using these algorithms can reduce the number of CRPA isolates that need to be tested by 40–50%. This effectively increases the yield of carbapenemase testing thus lowering the cost of testing by excluding low-risk isolates [16–18]. An assessment of the workflow and associated cost of molecular carbapenemase screening in *P. aeruginosa* is warranted to inform practice implementation.

## Methods

### Study site

**Figure 1.**
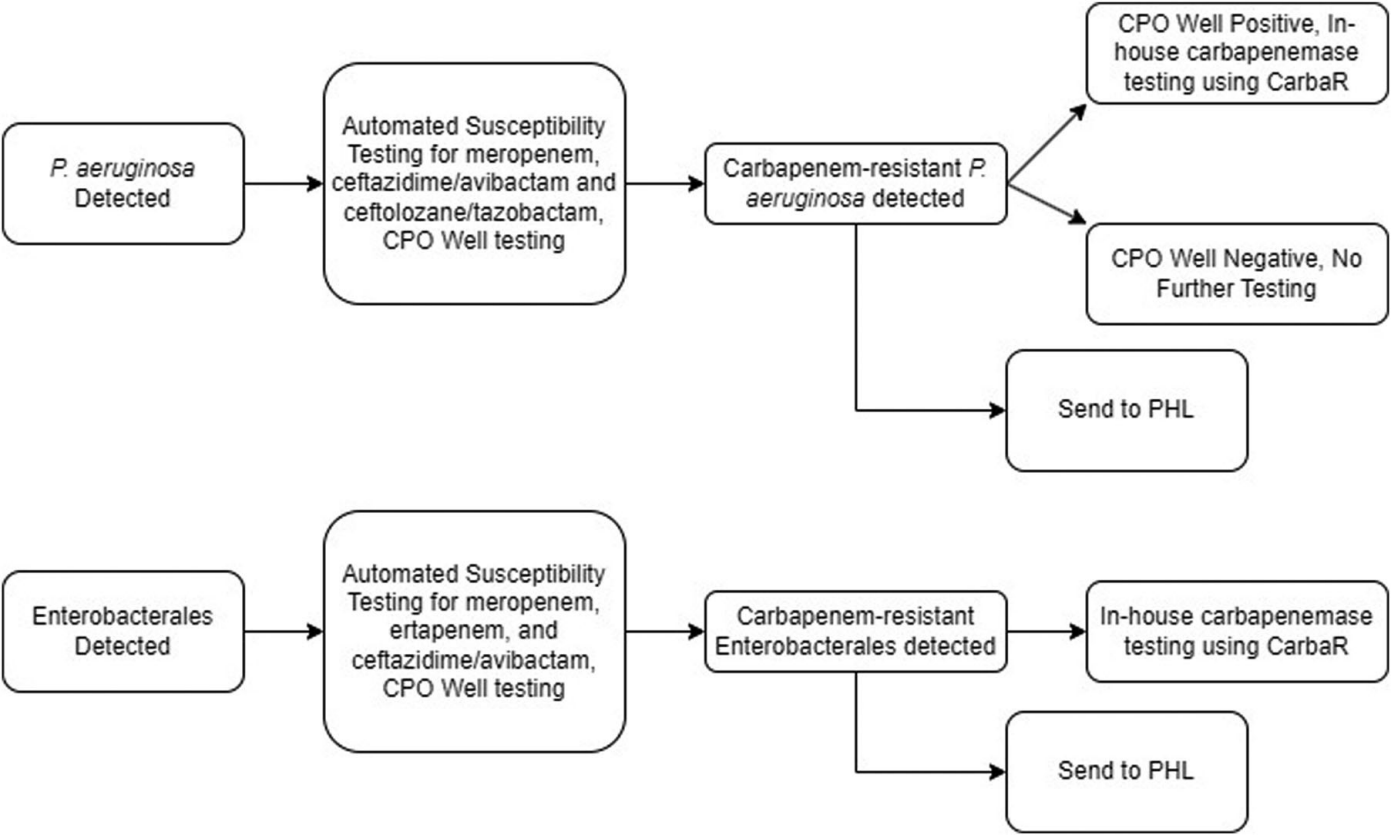
Current workflow for carbapenemase testing carbapenem-resistant *P. aeruginosa* isolates. Automated antimicrobial susceptibility testing was performed with BD Phoenix Platform. CPO well = the detection test for carbapenemase-producing organisms on the BD Phoenix panel. CRE = Enterobacterales resistant to either meropenem or ertapenem. CarbaR, PHL = public health laboratory.

The present study was an observational, time and motion assessment at a clinical microbiology laboratory using the established workflows for the detection and reporting of carbapenemases in targeted pathogens. The study was performed within the Hartford Healthcare Ancillary Microbiology Laboratory (HHAL) from May 2021 to July 2022. HHAL is a consolidated microbiology laboratory, performing work for seven hospitals. The standard laboratory workflow for carbapenemase testing for this study is depicted in Figure 1. Phenotypic antimicrobial susceptibility testing (AST) and molecular carbapenemase results are reported concurrently in the electronic medical record (EMR). Molecular carbapenemase testing was conducted per HHAL’s standard procedures for *P. aeruginosa* if the isolate is determined to be phenotypically carbapenemase-producing organism (CPO) well positive on automated susceptibility testing (Figure 1). Isolates of carbapenem-resistant *P. aeruginosa* in addition to CRE and CRAB isolates were submitted to the public health laboratory (PHL) for confirmatory phenotypic AST, supplemental mCIM, and carbapenemase gene testing. Results were returned to the clinical laboratory for review.

### Workflow assessment

To assess the time needed to complete carbapenemase testing with the Cepheid Xpert® Carba-R (Cepheid, Sunnyvale, CA, USA), microbiology technologists recorded the time required to complete hands-on tasks per standard operating procedures (SOPs) rounded to the nearest minute, excluding instrument run time. The Carba-R test detects the genes encoding KPC, NDM, VIM, OXA-48 and IMP. Methodology of testing isolates of *P. aeruginosa* and Enterobacterales are the same and both groups were included in the analysis to provide enough observational events for significance. The time needed to complete routine antimicrobial susceptibility testing (AST) was included. Routine AST is performed using the BD Phoenix automated system with corresponding Phoenix NMIC-306 Panel (Becton Dickinson and Co., Franklin Lakes, NJ, USA). Assessments were normalized for one isolate at a time. Technologists measured the amount of hands-on time needed to complete the send-out task as part of routine workflow.

### Time to carbapenemase test results

In-house testing turnaround times were compared to PHL send-out testing times. The date and the time the carbapenemase gene results were reported within the EMR were compared to the date and the time of electronic notification/fax receipt from the PHL.

### Cost analysis

Labor cost was estimated by multiplying the amount of time needed to complete the observed tasks by a national average estimate for microbiology technologist’s hourly pay (i.e. $38.11) per US Bureau of Labor Statistics[19]. The cost of Carba-R testing was estimated using fair market price ($421.58/ 10 tests) and cost to the laboratory per isolate was calculated ($34.18), which included supplies and shipping cost. Shipping cost was calculated for the study site but costs may vary in different laboratories (i.e. supply cost, shipping distance, etc.).

To estimate the cost of contact isolation incurred while carbapenemase production was ruled out, a published estimate of $158.10/patient-day was utilized[20]. The cost estimate was compared to the turnaround time for a negative carbapenemase gene test to extrapolate an estimate of the added cost of contact isolation. To estimate the number of carbapenem-resistant *P. aeruginosa* encountered per year, the 2021 inpatient antibiogram data for the HHC system hospitals was utilized.

## Results

 Twenty-eight observations of molecular testing were assessed. Normalized to testing of a single isolate, the median hands-on time of carbapenemases testing was 7.5 min (range 5–14 min). This was comparable to observed hands-on time for AST, which was a median of 10 min per isolate (range 8–20 min, *N* = 19). A total of 18 timed observations (*N* = 18) were conducted for sending isolates to the PHL. A median of 10 min of hands-on time was needed (range 8–22 min).

**Figure 2.**
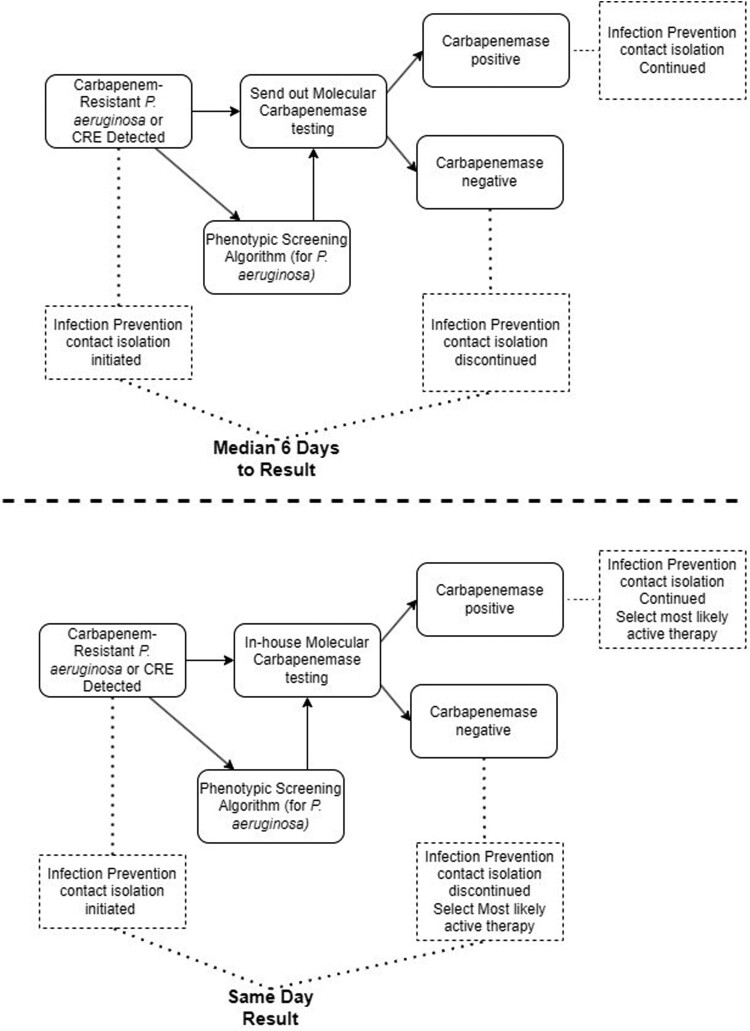
Standard Pathway PHL testing results in a median 6-day delay in receipt of carbapenemase testing results compared with same day results for in-house testing. This delays removal of patients from contact precautions while testing for carbapenemases is being conducted. The 6-day delay negatively impacts rapid therapeutic decisions especially if laboratories have implemented cascade testing. Same day carbapenemase testing can inform treatment decisions if cascade phenotypic susceptibility testing protocols are utilized, resulting in only a ∼24 h delay[19]. Supplemental phenotypic algorithms specifically to identify *P. aeruginosa* isolates that likely harbour carbapenemases may streamline testing in either scenario.

Current in-house laboratory practice is to perform the Carba-R test on the same shift as AST results become available. If meropenem is resistant and if the CPO test on the AST panel is positive, the Carba-R assay was automatically performed for *P. aeruginosa*. For CRE, all meropenem or ertapenem-resistant isolates were tested on the Carba-R regardless of CPO well result (Figure 1). Conversely, the elapsed time from detection of carbapenem-resistance by in-house testing for 66 isolate observations (N = 66) to the time results were available from the PHL Standard Pathway carbapenemases testing was a median of 6 days (range 3–14 days). Figure 2 displays the delays noted and the differences in actionable result times for carbapenemases when detected via in-house testing and results from the PHL. It must be noted that the CT DPH implemented a Fast Track Pathway for CRE and CRAB molecular carbapenemase detection in January 2022 for difficult-to-treat infections, expanding testing to 7 days per week. Assessing only isolates sent out after this implementation, the median time to result dropped to 5 days (range 3–7 days).

 In-house Carba-R testing is associated with an estimated cost of $46.92 per isolate, while the cost of sending out the isolate to the PHL was $40.53. The annual hospital antibiogram predicted 81 carbapenem-resistant *P. aeruginosa* would be recovered at a testing cost of $3,800.82, using Carba-R in-house versus $3,283.07 for send outs, annually. Considering the cost of contact isolation, delays associated with the median 6-day result turnaround time for the external assessment of carbapenemases, AST and molecular testing at the PHL is associated with additional cost of $948.60 per isolate or $76,836.60 annually.

## Discussion

Carbapenemase-producing Enterobacterales and *P. aeruginosa* are an increasing clinical challenge in the United States. The expanding prevalence of plasmid-encoded carbapenemases, such as KPC, VIM and NDM, are proving that the need for rapid detection of resistant organisms is paramount to optimize therapeutic strategies and infection prevention interventions. The present study demonstrated that in-house testing reduced the time to results from days to hours, when compared with send out carbapenemase testing at the PHL. This was accompanied by a limited increase in hands-on microbiologist time.

Early differentiation of the type of carbapenemases present in an isolate from an infection (i.e. serine versus metallo-β-lactamase) can aid clinicians in selecting their antimicrobial therapeutic strategies since carbapenemases many of the newer β-lactam/β-lactamase inhibitor combinations are not active against bacterial strains that harbour metallo-β-lactamases. Therapeutic pathways can be mapped by comparing phenotypic AST profiles to those of a specific carbapenemases gene, enabling selection of the antimicrobial agent most likely to be efficacious when a particular carbapenemase gene is detected[6,9]. Such strategies have been well established for treatment of carbapenemase-producing Enterobacterales[4], however; expansion of directed carbapenemase testing to *P. aeruginosa* may help guide treatment decisions. A global surveillance programme determined that ceftazidime/avibactam is highly effective against KPC-harbouring *P. aeruginosa*[6]. In contrast, detection of metallo-β-lactamases (i.e. NDM, VIM, etc.) is associated with near universal resistance to ceftazidime/avibactam and ceftolozane/tazobactam, necessitating clinicians to select alternative antimicrobial options[6]. The pairing of the carbapenem resistance phenotype with the specific carbapenemase detected (e.g. meropenem-resistance, NDM-positive) gives the physician a much clearer picture of which antimicrobial agents should be considered as viable options for therapy. Cascade susceptibility testing algorithms are commonly utilized in US health system clinical microbiology laboratories. These may indicate setup of additional antimicrobial agents to test by manual methods (e.g. disk diffusion or MIC test strip/gradient diffusion strips) or for automatic suppression or reporting of specific antimicrobial agents based on susceptibility profiles. However, this may delay reporting of results for an additional 24 h[21]. These study data indicate that implementation of genotypic carbapenemase testing may provide actionable information on the same day that the phenotypic carbapenem-resistance data are known by detecting or ruling out common carbapenemase genes in Enterobacterales and *P. aeruginosa*. These advantages will continue to evolve as the carbapenemase detection spectrum broaden including Guiana Extended Spectrum (GES)-carbapenemases, which have been noted in *P. aeruginosa* both globally and in the United States [6,16,22,23].

Rapid carbapenemase detection can also impact selection of infection prevention measures. Due to the transmissibility of plasmid-mediated carbapenemases, outbreaks are an important clinical concern particularly in the context of the National Healthcare Safety Network (NHSN) monitoring of all antimicrobial agent use and antimicrobial resistance in US Hospitals, which will be mandatory effective from 2023. The presence of plasmids carrying carbapenemases heightens the need for effective control measures relative to less resistant isolates as such mechanisms can result in little to no available treatment options in the setting of infection.

This study determined that same day in-house carbapenemase gene testing provided an actionable result much earlier than the PHL Standard Pathway (median 6 days). This can streamline infection prevention transmission strategies by allowing removal of contact precautions several days earlier by ruling out the majority of common carbapenemase genes. Contact precautions are recommended by the CDC for patients colonized or infected with CRE, however; not for CRPA[24,25]. Although, carbapenemase-producing *P. aeruginosa* are listed as a Tier 2 organism where contact precautions are indicated based on recommendations from the 2017 updates to the Interim Guidance for a Public Health Response to Contain Novel or Targeted Multidrug-resistant Organisms (MDROs) by the CDC[26]. Thus, the strategy of initiating contact precautions when CR-PA is detected is reasonable until carbapenemases are ruled out in which case discontinuation may be appropriate. Previous data have suggested that contact precautions, as a component of multimodal infection prevention bundles, have successfully stopped transmission of CP-CRPA though data for non-carbapenemase-producing organisms is lacking [27–29]. The discontinuation of contact precautions based on risk models may also improve clinical care, as contact precautions have been associated with poor patient experience and satisfaction[30]. Future studies should assess implementation of carbapenemase gene-based testing to guide infection prevention practices for interrupting transmission.

Even though this study suggests that the cost and labour of in-house carbapenemase testing is similar to the practice of PHL antimicrobial resistance testing, the real benefit to patient care is the improved turnaround time noted by performing carbapenemase gene testing in-house. Cost limitations to universal testing are recognized, particularly in the United States, where carbapenemase-producing *P. aeruginosa* remains relatively less prevalent compared with Enterobacterales where the majority of CRE harbour carbapenemases[1,6,8]. Strategies to streamline carbapenemase-testing in *P. aeruginosa* have been published and validated in regions with increasing prevalence of carbapenemase-producing *P. aeruginosa*[16–18]. Limiting carbapenemase gene testing to *P. aeruginosa* isolates that meet the following phenotypic algorithm (resistant to either meropenem or imipenem and non-susceptible to cefepime and ceftazidime) has been shown to significantly reduce molecular carbapenemase testing, while maximizing detection of carbapenemase producers[16–18]. Most commonly utilized antimicrobial agents may undergo initial AST with reflex to newer agents (i.e. ceftolozane/tazobactam or ceftazidime/avibactam) only occurring if the initial carbapenem result is resistant, potentially delaying interpretation of targeted therapy susceptibility results[21]. Further refinement of the phenotypic testing algorithm by adding secondary criteria of ceftolozane/tazobactam-non-susceptible can improve the algorithms test performance if centres have this information readily available. Utilizing the aforementioned phenotypic AST algorithm (meropenem or imipenem-resistant + cefepime and ceftazidime-non-susceptible) to determine if molecular carbapenem testing should occur, can reduce molecular testing by 40–50%, depending on isolate population, improving cost-effectiveness of implementing CRPA molecular testing.

The presence of phenotypic CPO detection methods on automated susceptibility panels may be a suitable screening tool to guide molecular carbapenemase testing efforts since it is FDA approved for use in *P. aeruginosa*. Such strategies may be useful in streamlining molecular testing as the CPO well testing has high sensitivity and negative percent agreement (97% and 96%, respectively) for *P. aeruginosa* [31,32]. Although false-positive results have been reported for *P. aeruginosa*, the positive percent agreement remains high (93%) for this species [31–33]. This method may be advantageous as it is also completed simultaneously with carbapenem MIC testing, however; validation in organisms with emerging carbapenemases (i.e. GES) is warranted as other phenotypic screens have failed to consistently detect GES-harbouring *P. aeruginosa* [31–36]. Similarly, molecular carbapenemase testing with the Carba-R is also approved for use in *P. aeruginosa*. Indeed, although analysis was pooled for Enterobacterales, *Acinetobacter* sp., and *P. aeruginosa,* the assay resulted >98% sensitivity and specificity in detecting NDM, VIM, IMP, OXA-48, and KPC[37]. Specific to *P. aeruginosa*, previous studies have found high test performance especially for VIM-harbouring isolates which make up the majority of CP-CRPA, although; future iterations of the assay may fill gaps in detection spectrum including rare IMP-variants and GES-type β-lactamases further expanding the utility of the test [22].

In conclusion, extension of in-house carbapenemase testing to *P. aeruginosa* can provide actionable results sooner than relying on send out testing. The present study provides data assessing the labour and cost of such strategies, showing limited hands-on time are needed for implementation. Rapidly ruling out carbapenemase production can have implications for both therapeutic decisions and infection prevention efforts. Future studies should assess the impact of implementation of such strategies on clinical management.

## Ackowledgements

We would like to acknowledge all of the members of the Hartford Healthcare Microbiology Laboratory for their technical support, especially Lori Baumgartner.
